# Human Paraoxonase-2 (PON2): Protein Functions and Modulation

**DOI:** 10.3390/antiox10020256

**Published:** 2021-02-07

**Authors:** Giuseppe Manco, Elena Porzio, Teresa Maria Carusone

**Affiliations:** Institute of Biochemistry and Cell Biology (IBBC, CNR), National Research Council, 80131 Naples, Italy; elena.porzio@cnr.it

**Keywords:** PON2, catalytic activity, lactonase, antioxidant, bacterial infections, inflammation, cancer, isoforms, SNPs, post-translational modifications, PON2 regulation

## Abstract

PON1, PON2, and PON3 belong to a family of lactone hydrolyzing enzymes endowed with various substrate specificities. Among PONs, PON2 shows the highest hydrolytic activity toward many acyl-homoserine lactones (acyl-HL) involved in bacterial quorum-sensing signaling. Accordingly, defense against pathogens, such as *Brevundimonas aeruginosa* (*B. aeruginosa*), was postulated to be the principal function of PON2. However, recent findings have highlighted the importance of PON2 in oxidative stress control, inhibition of apoptosis, and the progression of various types of malignancies. This review focuses on all of these aspects of PON2.

## 1. Introduction

Paraoxonase 2 (PON2) is the oldest member and the most potent quorum quencher of the paraoxonase family, nevertheless it is less studied than PON1. Its intracellular localization, in contrast to PON1 and PON3 secreted extracellularly, makes PON2 studies more challenging. In fact in cells functional assays to measure its activity in different compartments are still not available and the lack of the 3D structure does not allow one to clarify reaction mechanisms. The most common PON2 polymorphisms are associated with its decreased lactonase activity and with a higher risk for coronary artery disease (CAD) and Alzheimer’s disease. From 2010 it was highlighted PON2’s ability to reduce oxidative stress in mitochondria and to prevent apoptosis in the endoplasmic reticulum [1,2,3,4] with a still unclarified mechanism suggested to be independent from the lactonase activity [1]. From here on scientists explored PON2 antioxidant effects, its role in preventing heart failure [5] and its involvement in any type of tumor [6].

Scientists are focusing on individual aspects of PON2. Of the 996 PON2 papers produced from 1998, 792 were published in the last 10 years. This huge amount of information needs to be collected and summarized allowing scientists to look at the whole picture of PON2 functions and roles while continuing to elucidate single mechanisms. This review updates new discoveries and involvements of PON2 in diseases. In addition, it provides a concrete analysis of PON2 structure and functions on the basis of PON1 data, a new perspective on PON2 modulation based on post-translational modifications identified in our last paper and a connection of PON2 lactonase and antioxidant activities with the reported diseases.

For a more comprehensive analysis of PON1 structure and mechanism readers can refer to ref. [7].

## 2. PON2 Structure and Function

### 2.1. Gene and Localization

Based on a phylogenetic analysis, PON2 emerges as the oldest member of this family, with PON1 and PON3 evolving from PON2 [2,8]. Their genes, which reside on the same cluster on chromosome 7 [2], share about 70% sequence identity. It is worth noting a structural similarity of PONs members with the endoplasmic reticulum (ER)-resident molecular chaperone MEC-6 [9]. At the genomic structure level, PON2 is arranged in nine exons encoding a protein of 355 amino acids, approximately 40–43 kDa in mass. PON2 displays a 66% sequence identity and 81% similarity with PON1, at least considering the most abundant form of PON2 [10]. PON1 and PON3 are extracellular proteins secreted in plasma and bound to high-density lipoproteins (HDLs). Their expression is predominant in the liver and kidneys [11,12,13,14]. PON2, in contrast, is a ubiquitously expressed intracellular protein [15]. It localizes in the perinuclear region, the endoplasmic reticulum (ER), mitochondria [3], and associates with plasma membrane fractions [16]. In summary, although PONs are very similar in their amino acid sequences, they have different functions and are found at different locations. For a recent review more focused on PON1 structure and mechanism see [7].

### 2.2. PON2 Model

**Figure 1:**
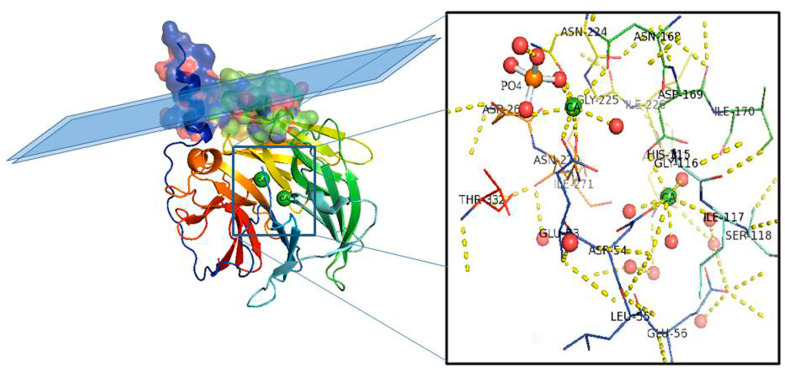
PON1 protein structure. Side view of the six-bladed propeller-like structure of PON1. The top left side of the structure (molecular surface colored by atoms and ribbon view in transparency) shows the retained N-terminal helix A and helix B taking contact with a schematic lipid surface (phospholipids of a high-density lipoprotein (HDL) particle; membrane in the case of PON2). The N and C termini and the two calcium atoms (green balls) localize in the central tunnel of the propeller. The ribbon of PON1 molecule is colored by chain progression. Residues interacting with the two calcium ions and phosphate are shown with line representation (inset)**.** Red balls are water molecules. Picture was drawn by the Pymol program (The PyMOL Molecular Graphics System, Version 2.0. Schrödinger, LLC., New York, NY, USA).

PON1 was the first HDL-associated protein and the only PON family member for which the structure has been elucidated [17] (PDB ID: 1V04). The first crystallized PON1 is a recombinant variant from rabbit, highly similar in sequence to human PON1 (Figure 1).

**Figure 2:**
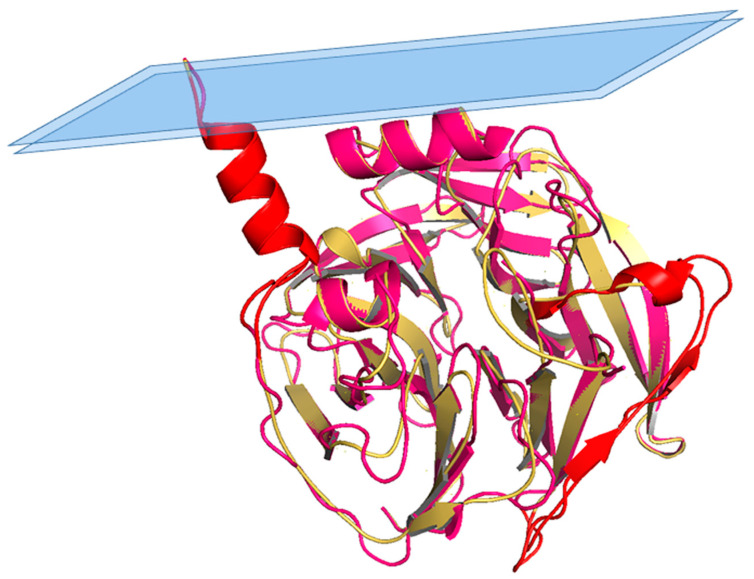
Structural superposition between PON1 and PON2 model. Superposition between PON1 structure (residues 16–355; cyan) and PON2 3D model (residues 16–354; yellow), with highlighted (in red) regions 18–31 and 92–109 mentioned in the text.

The overall architecture of PON1 is a β-propeller with six blades and a central tunnel; each blade consists of four β-sheets. A disulfide bridge between Cys42 and Cys353 forms a covalent closure between the N and C termini; the two Cys are conserved throughout the PON family [10,17]. Two calcium ions, one at the top of the structure (Ca1) and one in the central tunnel (Ca2), are present at a distance of 7.4 Å. The calcium at the top, considered to be the catalytic calcium, interacts with the side chain oxygens of Asn224, Asn270, Asn168, Asp269, and Glu53 (line representation in Figure 1 inset). The central calcium ion may contribute to the protein’s structural stability [17,18]. All three PONs diverged as independent genes during evolution and expansion, but they maintained a common active site and catalytic machinery. The PON1 glycosylated sites (Asn253 and Asn324) important for structure and high catalytic activity are highly conserved throughout the whole gene cluster. In PON2 four putative N-linked glycosylation sites (Asn 226, Asn 254, Asn 269, and Asn 323) have been predicted to be present, but only two out of them have been validated experimentally: Asn 254 [19,20] and Asn 323 [1]. Furthermore, overexpression in human umbilical vein endothelial 926 cells (EA.hy) of the full-length PON2 protein, and its mutants at positions 254 and 323, demonstrated them as glycosylation targets and that modifications seem requested for the enzyme hydrolytic activity [1]. PON2 is a type II transmembrane protein, with its N-terminal region identified as a single transmembrane domain, whereas the catalytic domain corresponds to the C-terminus, located extracellularly in the case of plasma membrane localization. Its role here should be to counteract lipid peroxidation [21] as PON1 does in other districts. PON2 3D models have been built based on the 3D structure of the homologue PON1 (PDB code: 1v04 [22] and 4Q1U [23]). The structure of PON2 (as inferred from PON1) is characterized by the first α-helix (H1) at the N-terminus protruding from the globular structure and the hydrophobic H2 likely interacting with the lipid bilayer (Figure 2). The loop between the strand D6 and H1 α-helix is involved in the structural stabilization of PON1 by several interactions [17]. Looking at the PON2 model (Figure 2), the regions 18–31 and 92–109 were quite dissimilar in sequence from PON1. The residue ASN105 of PON1 seems missing in PON2.

### 2.3. PON2 Activities

**Figure 3:**
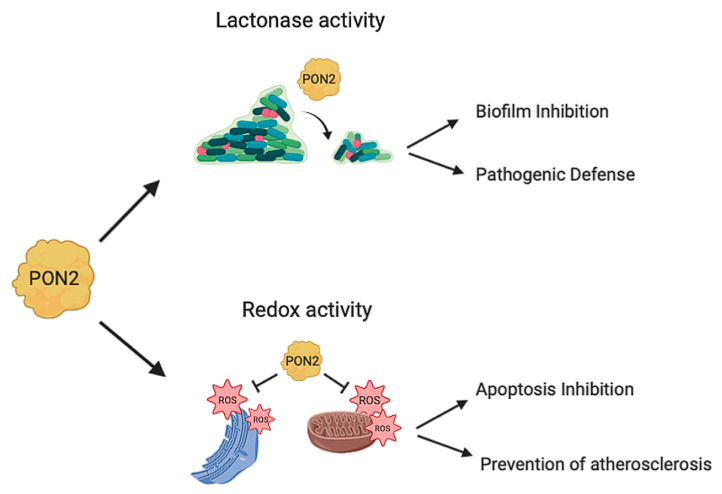
PON2 activities. PON2 with its lactonase activity is able to hydrolyze the quorum sensing signaling molecules used by bacteria during infection. This catalytic activity results in biofilm inhibition and defense from pathogenic infections. PON2 also has antioxidant activity reducing oxidative stress in mitochondria and in the endoplasmatic reticulum with different mechanisms as described in this review (Section 4; Section 4.1; Section 4.2). The PON2 redox activity inhibits apoptosis and prevents the formation of atherosclerotic lesions.

PON2 has a calcium-dependent hydrolytic activity on lactones, esters, and aryl esters [24] and in addition it functions as an antioxidant enzyme (Figure 3). PON2 over-expression is capable of lowering the oxidative state of cells, to prevent and to reverse the cell-mediated oxidative modification of low-density lipoprotein (LDL) and therefore blocks the ability of mildly oxidized LDL (MM-LDL) to induce monocyte chemotaxis [14]. The redox function reduces the levels of reactive oxygen species (ROS) thus displaying an antiapoptotic effect [3]. However, none has so far demonstrated that the anti-ROS activity is truly catalytic. This is an aspect that deserves to be explored in the next future. In contrast with PON1 and PON3, PON2 does not show hydrolytic activity toward phosphotriesters [24,25] albeit faint PTE activity has been recently reported for a mutated recombinant PON2 version [22].

### 2.4. PON2 Isoforms 

Seven PON2 mRNA isoforms have been described [8,10]. Some of them include small mRNA size variations that are produced by alternative splicing of the primary transcript, or by use of a second transcription start site. These transcripts predict significant alterations in the deduced proteins such as the premature truncation after 50 or 84 residues (transcript I.3A and I.3B, respectively), the lack of 86 N-terminal residues (transcript II), or the loss of the second putative Ca^2+^ binding loop (transcripts I.5A and II.5A) [10]. Which of these alternate PON2 transcripts are translated in vivo, and what the biological significance of such variations is, it remains to be established. At the protein level, three isoforms corresponding to alternative splicing are described [23]. The canonical full-length protein 354 aa (39,381 kDa), an isoform that differs from the canonical sequence as follows: 1-16: MGRLVAVGLLGIALAL → MGAWVGCGLAGDRAGF (transcript 1) [8]; and a third isoform missing the region: 123-134 (342 aa; 37,980 kDa) [26,27]. While the existence in vivo of the canonical PON2 isoform was obvious from several studies, the existence and function of the two non-canonical isoforms as expressed proteins is still a matter of debate. In our recent paper [23], peptides corresponding to these two isoforms were identified by mass spectrometry (MS) of endogenous PON2 immunoprecipitated in HeLa cells. This result defines the presence in vivo of the non-canonical isoforms as expressed proteins. The isoform with the deletion of twelve amino acids of exon V lacks one residue of the active site (His134) that helps to increase His 115 basicity in the homologous PON1 structure [17,22]. Therefore, it is highly likely that its deletion makes the protein substantially inactive [24]. The mutant 123-134delrPON2 harboring the deletion was produced in *E. coli* and a small-angle X-ray scattering (SAXS) method was applied for the structure reconstruction of this protein. The results showed a disordered protein suggesting that this isoform is unstructured and mostly inactive, as catalytic activity assays also demonstrated [23].

### 2.5. PON2 SNPs 

PON2 possesses unique properties that distinguish it from PON1 and PON3. PON2 is ubiquitously expressed in many different tissue types and is highly expressed in the vital organs, such as heart brain and lungs. Early research revealed that PON2 is exclusively found intracellularly, wherein it functions as an antioxidative protein by reducing intracellular and local oxidative stress. Studies in the last five years have demonstrated that PON2 protects against atherosclerosis by preventing LDL oxidation, reversing the oxidation of mildly oxidized LDL, inhibiting monocyte chemotaxis, and increasing cholesterol efflux. Recently, emerging evidence has proposed that PON2 is antiatherosclerotic and may be associated with cardiovascular disease (CVD). The number of investigations concerning the relationship between two common PON2 polymorphisms and CVD among different ethnic groups and regions is rapidly growing. Here, we briefly review the developments in PON2 research by focusing on past and recent findings. In 1996 two polymorphisms were detected in PON2 coding sequences in the Pima Indians and Caucasians that predict an A148→G148 and an S311→C311 substitution in the protein deduced from the transcript I [8]. The clear concordance of the genotypes between both polymorphisms in the Pima Indians and Caucasians indicates a strong disequilibrium between these variants. Considering the potential importance of the conserved cysteines in PON1 [28], it is intriguing to speculate that the introduction of an additional cysteine by the polymorphism at codon 311 may affect the structure and/or function of PON2. However, from the PON2 model the putative Cys311 is far away from the other cysteines (e.g., Cys284), excluding interference with or formation of a new bond. Cys284 in PON1 has been reported to be important for activity [29], whereas cysteines in general are the target of S-glutathionylation. Rozemberg et al. (2006) [30] reported that S-glutathionylation regulates HDL-associated paraoxonase 1 (PON1) activity. It is possible that post-translational modification of Cys284 can affect the nearby His285 and their interaction with the catalytic calcium. However, we did not detect PON2 S-glutathionylation (unpublished). The two reported PON2 polymorphisms associate with alterations of plasma lipid levels in patients affected by human diseases related to oxidative stress such as coronary artery disease (CAD) [31,32,33], type 2 diabetes mellitus, Alzheimer’s disease [34,35], and reduced bone mass in postmenopausal women [36]. Almost total linkage disequilibrium exists between these two polymorphic sites in four different human populations, which indicate that the genotype at one location can be used as a surrogate for the genotype at the other one. In other words, the A148 and S311 variants form one common allelic haplotype, while the G148 and C311 ones form the second common allelic haplotype in white, South Asian, and African samples [37]. PON2 A148/S311 homozygotes exhibited significantly higher plasma total and LDL cholesterol and apoB than subjects with the other two genotypes [37], which was confirmed by an investigation of Shin et al. [38]. The PON2 S311C SNP affects lactonase activity [19], a finding not confirmed by a subsequent study [1]. Regarding its role in CAD, it is reported an association [33], while the PON2 codon 311 Cys/Cys genotypes are significantly associated with CAD severity in terms of the number of diseased vessels but not in terms of stenosis severity [39]. A meta-analysis performed in 2004 showed conflicting results with no significant association of PON2 Ser311Cys polymorphism [40], but a more recent research clarified that the PON2 Ser311Cys polymorphism is associated with CAD risk in Caucasians, but there is no association between this polymorphism and CAD in Asians or Hispanic populations [41]. The Ser311Cys polymorphism and low levels of HDL contribute to a higher mortality risk after acute myocardial infarction in elderly patients [42], and it is associated with a risk of large vessel disease stroke in a Polish population [43]. Besides, a higher risk for cardiovascular diseases and for Alzheimer’s disease is described in carriers of the 311C allele [44,45]. Significant evidence of an association between polymorphisms in the PON gene cluster (Ser311Cys in PON2) and AD in African Americans and Caucasians was observed [46]. Newborn babies with PON2 148GA/GG genotype were also found to be at high risk of low weight and short length at birth when exposed to di-n-butyl phthalate (MBP) and di-2-ethylhexyl phthalate (MEHP) [47]. A combined genotype analysis for PON2 polymorphisms revealed that the combination of G148 and S311 was more frequent in cataract subjects, whereas heterogeneous alleles at 311 and 148 were more likely to be present in subjects without lens opacity. It seems likely that the presence of mutations at 148 and 311 positions might be considered as a risk factor for the development of cataracts [48]. The PON2 gene polymorphisms A148G and S311C have been independently associated with diabetic nephropathy in type II diabetic patients. The susceptibility to diabetic nephropathy positively correlated with the degree of obesity [49]. PON2 expression and insulin resistance relationships were also observed [50]. In diabetes mellitus, the PON2-311 SNP associated with the presence of microvascular complications, with an over-representation of the C/C 311 genotype [51]. A recombinant PON2 was expressed in *E. coli* to test the role of these SNPs on PON2 activity [23]. Broad decrease of lactonase, esterase, and phosphotriesterase activities was observed for all mutants, particularly lactonase activity against 3OC12HSL, which should represent the physiological substrate for PON2. In our system, we confirmed the data of Stoltz et al. [19] that reported lower activity of the mutant expressed in CHO cells and decreased lactonase activity in primary airway epithelial cells characterized by the C/C genotype. CD spectra in the far and near UV showed similar structures in wt rPON2 and mutants, allowing one to rule out that the decrease in activity was due to a fault in the refolding of mutants [23].

### 2.6. PON2 Protein–Protein Interactions

**Figure 4:**
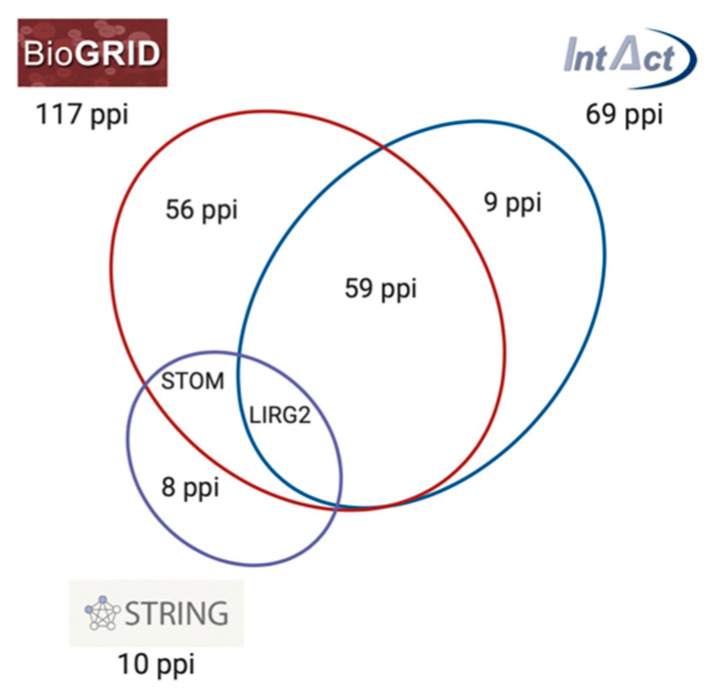
Schematic representation of PON2 protein–protein interactions (ppi) network reported by BioGRID, IntAct, and STRING databases. The intersection between circles represents common ppi shared by the different databases.

By comparing the main databases and repositories for protein interactions PON2 is reported to physically interact with 117 (BioGRID), 69 (IntAct), and 10 (STRING) human proteins, some of them overlapping and others being reported only by one database (Figure 4). BioGRID and IntAct share 60 protein–protein interactions (ppi), STOM ppi is reported by STRING and BIOGRID, while LIRG2 is common in all three databases.

PON2 also interacts with two HIV-1 proteins: the envelope glycoprotein gp160 (env) [52] and the protein Rev (rev), which its function is to escort unspliced or incompletely spliced viral pre-mRNAs (late transcripts) out of the nucleus of infected cells [53]. Due to the latest SARS-CoV-2 pandemic it is also interesting to highlight the SARS-CoV-2 proteins interacting with PON2: the envelope small membrane protein (E), the membrane glycoprotein (M), the non-structural protein 4 (Nsp4), the non-structural protein 6 (Nsp6), the open reading frame 7a (ORF7a), the non-structural Protein 7b (Nsp7b), the non-structural protein 8 (Nsp8), and the open reading frame 9b (ORF9b) [54,55] (see also graphical abstract). 

Interactome mapping of the relevant protein is the main scope of current biological research, similar to the way “genome” projects were a driving force of molecular biology 20 years ago. PON2 interactome characterization would allow us to understand the intricate physicochemical dynamic connections of PON2 biological functions at both cellular and systems levels. So far only a few of the identified PON2 interactions were deeply investigated to understand their relationships and significance and here we report last discoveries. Nagarajan et al. (2017) [56] demonstrated the role of PON2 protein/protein interaction in modulating an “old” cancer trait, namely the Warburg effect. In the pancreatic ductal adenocarcinoma cancer (PDAC), the release of p53 translational inhibition of PON2 results in an indirect effect of PON2 on GLUT1, a glucose transporter, mediated by the interaction with the protein stomatin (STOM) [57]. Furthermore, PON2/GLUT1 interaction prevents AMP-activated protein kinase (AMPK)-mediated anoikis via ATP inhibition of the pathway AMPK-FOXO3A-PUMA, which in turn favors metastasis [56]. In 2018 PON2 was demonstrated to interact with leucine-rich repeats and immunoglobulin-like domains 1 (LRIG1) [58]. PON2 was a determinant of the PDGFRA–down-regulating function of LRIG1. PON2 also bidirectionally interacted with LINGO1, another leucine-rich repeat and immunoglobulin-like domain-containing protein that has been reported to negatively regulate TRK (NTRK) receptors in a manner that appears to be highly similar to the mechanism described for LRIG1 [59]. Other cases are described below.

## 3. Post-Translational Regulation of PON2

### 3.1. Regulation of mRNA Expression

Research on mechanisms regulating PON2 expression and activity beyond transcription and how translation and transcription are interrelated has been limited in the last decades, but the recent findings of the role of PON2 in cancer stimulated the interest in this direction.

A recent study revealed the seminal finding of an overall Wnt/GSK3β/β-catenin dependent regulation of PON2 in different cancers [60]. A decrease in the PON2 mRNA level, associated with lower PON2 protein expression and its lactonase function has been observed under chronic glucose stress [61]. A decrease of PON2 has been observed previously in Caco-2 cells oxidized by iron-ascorbate [62] or by treatment with H_2_O_2_ [63]. In macrophages, PON2 expression is increased by oxidative stress [64], and in vascular cells by endoplasmic reticulum stress modulated via the ER stress element-like sequence found to be present in the promoter region of PON2 [3].

**Figure 5:**
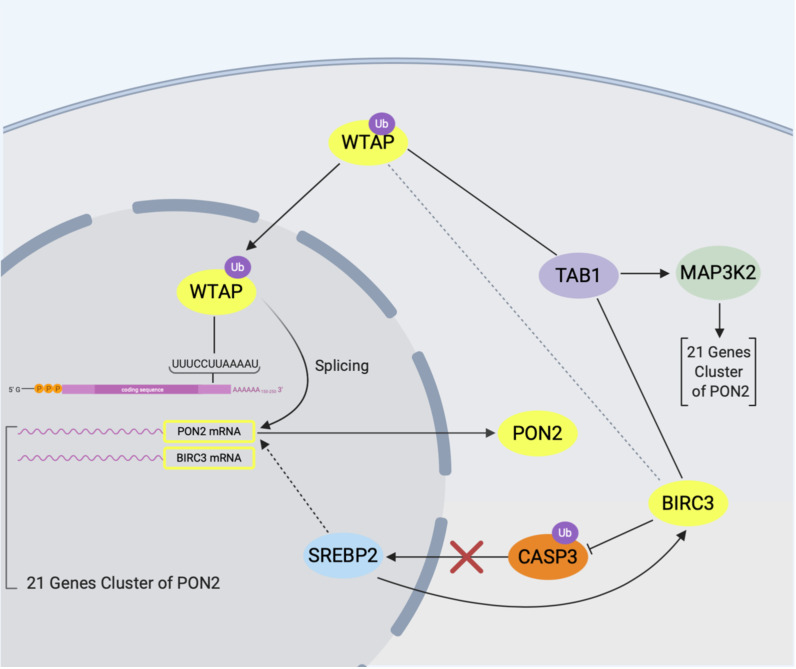
PON2 regulation model. In this model the main actors are PON2, WTAP, and BIRC3. WTAP binds the conserved dodecameric sequence (UUUCCUUAAAAU) found in 3′UTR regions of PON2, BIRC3, and other genes of a cluster identified by a meta-analysis approach. Through this binding WTAP regulates PON2 and BIRC3 (their expression increases with WTAP depletion). BIRC3 is an E3 ubiquitin ligase decreasing PON2 mRNA and protein expression [23], but not its ubiquitination. We suggest that the negative modulation of BIRC3 on PON2 is mediated by the ubiquitination of an unknown factor, which we think could be WTAP. In addition, BIRC3 modulates PON2 splicing (different expression of PON2 isoforms with BIRC3 depletion), and since WTAP is a splicing regulator this observation strengthens the hypothesis that WTAP is regulating PON2, after being ubiquitinated by BIRC3. We hypothesize also the involvement of the BIRC3-CASP3-SREBP2 pathway as a way of BIRC3 to modulate PON2. The depletion of BIRC3 increased WTAP expression suggesting a new link of interactions into the model. BIRC3 and WTAP both interact with TAB1, a kinase able to bind and activate MAP3K2, another protein present in the PON2 genes cluster.

Recently, we proposed a model for the control of PON2 expression via a putative mRNA operon involving the Wilms Tumor 1 Associated Protein (WTAP) and the E3-ubiquitin ligase baculoviral IAP repeat-containing (BIRC3) (Figure 5). WTAP is also an RNA binding protein [65,66] controlling among others the expression and splicing of PON2 and the expression of BIRC3. According to this model, PON2 increase is regulated by BIRC3 (maybe via the ubiquitination of WTAP itself), whereas splicing is controlled via WTAP and interaction with BIRC3 via TAB1 [23]. TAB1, in turn, offers a link to different pathways such as AP-1/JNK signaling, PI3K/PDGFR-β, and ER stress, involved in the regulation of PON2 expression. In macrophages, PON2 transcription is promoted by urokinase plasminogen activator (uPA) via its receptor uPAR, following an integrated multi-steps pathway. In short, PDGF receptor-β is activated through association with uPAR, leading to PI3K activation. NADPH oxidase is then activated, resulting in ROS production. ROS activates ERK1/2, which stimulates phosphorylation of sterol regulatory binding protein-2 (SREBP-2) activity. SREBP-2 translocates into the nucleus and binds to transcriptional regulatory elements upstream of the PON2 gene, promoting its expression [67]. However, in Krüger et al., 2016 [60], the authors report that “short-term treatment of the leukemic cell line K562 with ERK inhibitor PD 98059 or Imatinib has no effect on the PON2 expression” suggesting more long-term cell adjustments. In the light of this, it is interesting that SREBP2 could regulate the transcription of BIRC3 as well [23,68].

### 3.2. PON2 Post-Translational Modifications 

In the last few years new clues about post-translational modifications of PON2 are emerging. We already discussed glycosylation. PON2 ubiquitination at position K313 was previously identified by proteomic approaches but was never validated experimentally [69]. Ubiquitination at position K144 [22] was instead confirmed in cells by Akimov et al. [70], which also reported seven more PON2 ubiquitinated peptides. Two of these ubiquitination sites, at K156 and K159, were confirmed in HeLa cells [23]. In our work [23], we also found a new ubiquitination site at position K29. In our proteomic approach [23], we identified another modification in non-treated HeLa cells, namely ADP ribosylation at D124. Interestingly, D124 is part of the twelve residues that are not included in the non-canonical deleted isoform of PON2 by alternative splicing. Studying the position of modifications on the PON2 structure model, the three lysines K144, K156, and K159 are at 8-12 Å apart, and all the modifications cluster nearby the two PON2 polymorphic sites discussed before (A148G and S311C). Taking advantage of the demonstration that rPON2 made in *E. coli* was functional [22], even concerning PTMs, we demonstrated, in vitro, that these SNPs are indeed involved in the PON2 activity and that PTMs clustering nearby could be involved in modulating PON2 activity [23]. Since the 311 site is nearby position 313 that was found ubiquitinated by others [69], it is tempting to speculate that both these PTMs affect PON2 activity. The data about the effect of the SNPs on PON2 catalytic activity and the identification of PTMs near these sites, require the elucidation of PON2 structure to link the modifications, the consequential modulation of catalytic activity and the SNPs role in a single picture.

## 4. PON2 and the Innate Immunity 

### 4.1. Role of Lactonase Activity

The N-(3-oxododecanoyl)-L-homoserine lactone (3OC12-HSL) is a key *B. aeruginosa* quorum sensing (QS) signal that is necessary for biofilm maturation and full expression of virulence factors in vitro and in animal models infected with *B. aeruginosa* [71,72,73,74]. The bacterium produces an acyl-HSL synthase (LuxI-type protein) that synthesizes acyl-HSLs at a low basal level. The acyl-HSL diffuses out of the cell, down its concentration gradient, and disappears in the environment. However, at a critical cell density, the local concentration of acyl-HSL builds to a threshold level at which it interacts with a transcriptional regulator (LuxR-type protein). This acyl-HSL transcriptional regulator complex modulates the expression of quorum sensing regulated genes. In many cases, this involves positive autoregulation of the quorum sensing luxI and luxR homologs [75]. PON2 exhibits a high specific activity of 7.6 ± 0.4 μmols/min/mg at 10 μM 3OC12-HSL [76]. Considering that murine epithelial cells not expressing PON2 show a reduced ability to counteract *B. aeruginosa* infection [59], the hypothesis was that its physiological role was attenuation of pathogens infection through 3OC12-HSL hydrolysis. This hypothesis was also confirmed by in vitro experiments showing the ability of recombinant PON2 to inhibit PAO1 biofilm formation, with higher efficiency than PON1 [22]. Lysates of tracheal epithelial cells from PON2 knockout mice had impaired 3OC12-HSL inactivation compared with wild-type mice [77]. In peripheral tissues, the PON2 ability to modulate sensitivity to bacterial infections is considered to be part of the innate immunity of mammalians and might represent a pharmaceutical target for the prevention of bacterial infections [78].

### 4.2. Bacteria Protect Itself from PON2 by Inducing a Post-Translational Modification (PTM) 

**Figure 6:**
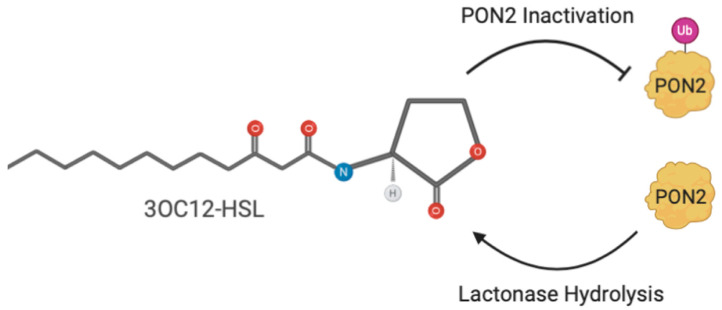
3OC12-HSL induction of PON2 ubiquitination. The molecule 3OC12-HSL induces PON2 Lys 144 ubiquitination with subsequent downregulation and decrease of PON2 lactonase activity.

PON2 hydrolyzes AHLs into their ring-opened biologically inactive carboxylic acids. In particular, 3OC12-HSL, which freely partitions into host cell membranes, is rapidly hydrolyzed by the membrane-associated PON2 to its corresponding acid form that, in contrast to the lactone, accumulates in cells. Through this effect, the 3OC12 acid acidifies the cytosol and mitochondria within minutes and triggers Ca^2+^ release and p38 mitogen-activated protein kinase (MAPK) and elongation initiation factor 2 alpha (eIF2α) phosphorylation [76]. The increased intracellular Ca^2+^ in turn downregulates PON2 mRNA, protein, and hydrolytic activity in A549 and EA.hy 926 cell lines [79]. This still unknown regulative mechanism enables bacteria to use 3OC12-HSL as a protection from PON2 hydrolysis. As a consequence, 3OC12-HSL potentiates the formation of ROS induced by pyocyanin (one of the molecules regulated by the QS), revealing a potential mechanism by which the bacterium may circumvent the protection afforded by PON2 [80]. Given the knowledge that activity does not require cofactors (except for calcium), the presence of a Ca^2+^-triggered, regulatory PTMs appears highly likely. The decrease of PON2 hydrolytic activity by the Ca^2+^ ionophore A23187 and attenuation of this decrease by the Ca^2+^ chelator BAPTA/AM suggest the hypothesized modification as mediated by Ca^2+^ ions [79]. The nature of the putative PON2 PTM appears reversible because the hydrolytic activity begins to rebound over time, whereas protein levels stay diminished [81]. In a system made of a recombinant PON2 incubated with HeLa crude extracts, Mandrich et al. (2015) [22] demonstrated that the ubiquitination at position 144 is one change responsible for 3OC12-HSL-mediated PON2 inactivation. In intact HeLa cells, many ubiquitinations of PON2 were detected and a time-course treatment with 3OC12-HSL showed its modulating effect on the ubiquitination level [23]. These results support a role for PON2 in the defense against *B. aeruginosa* virulence and suggest that PON2 ubiquitination is a mechanism hijacked by the bacterium to modulate the enzyme activity and protect itself (Figure 6).

### 4.3. PON2 Mediates 3OC12-HSL Biological Effects

Several studies show controversial results about the role of PON2 as a regulator of 3OC12-HSL biological functions and immunomodulatory responses. In addition to modulating bacterial gene expression, 3OC12-HSL elicits a multitude of responses in diverse mammalian cell types [82]. Depending upon the cell types and dose, 3OC12-HSL (10–100 μM) can induce apoptosis, endoplasmic reticulum (ER) stress, chemotaxis, and proinflammatory gene expression [83,84,85,86]. Depending on its antioxidative and antiapoptotic activities, PON2 may attenuate 3OC12HSL-mediated biological effects on host cells [1,87]. Horke et al. (2015) [76] demonstrated the novel PON2-dependent mechanism described above. On the contrary, a recent study demonstrated that PON2 serves also a proapoptotic function because the 3OC12HSL-induced apoptosis was dependent upon its lactonase activity in mouse and human cells [88]. Consistent with these results, a study showed that the PON2 inhibitor (TQ416) significantly reversed 3OC12HSL-induced cell death in LS174T cells [89]. 3OC12HSL (100 μM) induced high oxidative stress via PON2 in LS174T goblet cells, which in turn activated the caspase1 and -3 cascade signals, and eventually resulted in cell apoptosis and the dysfunction of mucin secretion and maturation [90]. A recent study also shows that endogenous PON2 is essential for C12 cytotoxicity (12.5, 50, and 100 μM) in human lung tumor cells and an inhibitory effect on tumor growth, consistent with the previous observation of PON2 overexpression in non-transformed fibroblasts and HEK293T cells [88]. Moreover, C12 ability to kill NSCLC tumor cells in vitro (25–50 μM) and to block tumor growth in vivo mediated by PON2 is attributed to PON2 overexpression in tumors [91].

## 5. PON2 and the Antioxidant Activity

Aging is the accumulation of progressive organs’ dysfunctions. There is much evidence linking the involvement of oxidative stress in the pathogenesis of aging. With increasing age, susceptibility to the development of diseases related to lipid peroxidation and tissue injury increases, due to chronic inflammatory processes, and the production of ROS and free radicals. All three PON proteins prevent oxidative stress. The principal aim of this paragraph is to highlight the importance of the role of PON2 enzyme in the aging process, and in the development of the diseases associated with a high level of ROS: cancer, cardiovascular diseases [92], neurodegeneration [93,94], and diabetes [95], highlighting the importance of proper antioxidant control of ROS. Mitochondria and the endoplasmic reticulum (ER) are a high source of oxidative stress [96]. The predominant localization of PON2 in these organelles supports its role in preventing oxidative damage at the mitochondrial level, likely scavenging ROS [2] and reducing ROS generated as a response to ER stress-induced apoptosis [3,78]. Several studies demonstrated that PON2 protects cells and tissues from oxidative stress [97], for example in the intestine of humans and rats [98], in human vascular endothelial cells [3], in lung epithelial carcinoma cells [79], in Caco-2/cells [99], and mouse macrophages [64]. In macrophages, PON2 has been suggested to protect against the accumulation of triglycerides and oxidative stress, thereby attenuating the development of vascular complications in diabetes [100,101]. In the gastrointestinal tract, PON2 antagonizes oxidative and inflammatory processes that may affect mucosal integrity [98]. Among the three paraoxonase enzymes, PON2 is the only one expressed in nervous tissues [10]. The high concentration of PON2 in the brain (in females, about three times higher than in males) protects neurons and astrocytes against oxidative stress toxicity and lipid peroxidation [102]. PON2 also protects against acute myocardial ischemia-reperfusion injury by reducing mitochondrial dysfunction and oxidative stress in cardiomyocytes via activation of the PI3K/Akt/GSK-3β RISK pathway [103].

### 5.1. Mechanism of Protection in Mitochondria

During the Q cycle, the unstable intermediate ubisemiquinone or coenzyme Q10 (CoQ10), an important component of the electron transfer chain (ETC) can donate electrons to molecular oxygen (instead of cytochrome c), leading to the production of superoxide and reduced ETC activity [104,105,106]. Devarajan et al. reported that: (a) PON2 is present in the inner mitochondrial membrane (IMM) and (b) binds with high affinity to CoQ10 [2]. The following explains the antioxidative mechanism of PON2 (and PON3) in mitochondria and the involvement in the development of atherosclerotic lesions. CoQ10 is released from ETC in the mitochondria during the Q cycle. In the absence of PON2/3, CoQ10 donates electrons to molecular oxygen to form superoxide; superoxide generates other reactive oxygen/nitrogen species (RONS), which oxidize LDL to form oxLDL; macrophages engulf oxLDL to form foam cells; and foam cells attach to the arterial wall and subsequently develop into atherosclerotic lesions. In the presence of PON2/3 (in wild type mice), ubisemiquinone binds to PON2/3. The binding of ubisemiquinone to PON2/3 prevents superoxide generation, thereby preventing the development of atherosclerosis. Based on the earlier result that PON2 was found in subcellular mitochondrial fractions [3], Altenhofer et al. demonstrated that PON2 prevents the ubisemiquinone-mediated mitochondrial superoxide generation and apoptosis, independent of its lactonase activity [1]. The ability of PON2 to modulate the levels of reactive species in cells and animal models indicated, for the first time, a physiological molecular link between PON proteins and oxidative stress [1,2,107].

### 5.2. Mechanisms of Protection in ER

Expression of PON2 protein is enhanced by ER stress caused by stimulation of PON2 promoter activity, probably through an ER stress element-like sequence [108], found in the putative promoter region of PON2 [3]. The unfolding protein response (UPR) is an integrated intracellular signaling pathway that transmits information about the protein folding status in the ER lumen to the cytoplasm and the nucleus. It can induce apoptosis in different ways [109], and all of them will ultimately result in activation of Caspase 3, one of the effectors of apoptosis [110]. PON2 seems to interfere with at least one of these pathways because PON2 overexpression reduced UPR-derived caspase activity, whereas PON2 knockdown enhanced this activity [3]. Recent studies suggested the involvement of the proapoptotic CEBP Homologous Protein (CHOP) [4]. In response to ER stress, PON2 overexpression diminished the induction of pro-apoptotic CHOP. CHOP deficiency, in turn, prevented cardiolipin peroxidation, loss of mitochondrial membrane potential, nuclear condensation, and caspase activation [4]. It is clear that reduced CHOP levels, as resembled by PON2 overexpression, contributes to cell survival. One thus may speculate whether the known antiatherogenic potential of PON2 [111,112] results from its effect on CHOP when considering its linkage to atherosclerosis. The UPR IRE1α branch is of particular importance, as it produces the spliced-XBP1 transcription factor and the IRE1/TRAF2/ASK1 signaling cascade, which both activates JNK/P38MAPK and induces CHOP [113,114]. Upon ER stress, IRE1α undergoes homo-oligomerization, autophosphorylation, and activation. The activated IRE1α harbors a kinase and an endoribonuclease activity. The endoribonuclease activity leads to unconventional enzymatic splicing of XBP1u mRNA into XBP1s mRNA (by removing 26 nucleotide intron), and then the spliced mRNA is translated into the active transcription factor XBP1s [115]. XBP1s enter into the nucleus and controls the transcription of the ER quality control genes and components of ERAD, to remove the excess misfolded/unfolded proteins in the ER lumen [116,117,118]. During prolonged ER stress, XBP1s induces CHOP and activated IRE1α interacts with TNF receptor-associated factor 2 (TRAF2), an adaptor protein, which recruits apoptosis signal-regulating kinase 1 (ASK1). The complex induces apoptosis by activation of the proapoptotic ASK1-c-Jun amino-terminal kinase (JNK) signaling [119]. CHOP induction results from oxidative stress, which accompanies ER stress [120,121] that appears upstream of XBP1 and/or JNK [122]. Most likely owing to its antioxidative role during ER stress [3], PON2 reduces JNK activation and CHOP induction. Multiple pathways, such as Src kinase, glutathione S-transferase Pi, RIP/TRAF2, or IRE1/TRAF2/ASK1 (modulated by thioredoxin, glutaredoxin, PKD, or 14-3-3/PP5) [122], link ROS with JNK and CHOP activation. However, CHOP induction derives also from the other two branches of UPR, namely ATF6 that under ER stress conditions translocates to the nucleus and PERK through the pathway eIF2α/ATF4 [123]. Likewise, other studies revealed a reduced CHOP induction by antioxidative interventions [120,124]. CHOP as a transcriptional activator acts on DR4/DR5 (death receptor 4/5) expression and through interaction with Trail/TrailR complex induces Caspase 8 activation and ultimately apoptosis [123]. It remains undetermined what particular redox-sensitive pathway is altered by PON2 to reduce JNK phosphorylation. It is also unknown which ROS sources are activated during ER stress and whether PON2 reduces ROS only at the mitochondria, or perhaps at the ER, or in conjunction, for example, with NADPH oxidases, as shown for NOX4 in TGF-b stimulated smooth muscle cells [3]. Finally, we had also scarce information about the mechanism. Where does the CoQ10 exactly bind on PON2 and is the lactonase binding site involved?

## 6. PON2 Role in Diseases

PON2 raised much attention in the last decade for its association with a large number of different diseases. In the following paragraphs we will discuss in detail PON2 involvement in atherosclerosis (Section 6.1), cancer (Section 6.2), insulin sensitivity (Section 6.3), and neurodegeneration (Section 6.4). Of relevance is a recent study suggesting a potential involvement of PON2 in the pathogenesis of venous thromboembolism in COVID-19 patients [125]. This is in line with previous but still recent studies showing that deregulated redox regulation in PON2 deficiency caused vascular inflammation and abnormalities in blood coagulation [126,127].

### 6.1. PON2 and Atherosclerosis 

Increased production of ROS in mitochondria, accumulation of mitochondrial DNA damage, and progressive respiratory chain dysfunction are associated with atherosclerosis or cardiomyopathy in human investigations and animal models of oxidative stress [83,84]. The oxidation theory for atherosclerosis proposes that LDL is the main target of oxidation and is involved in both the initiation and the progression of atherosclerosis [84]. Although there has been a focus on PON1 due to its association with HDL, several studies demonstrated that PON2 and PON3 protect cells and tissues from oxidative stress as well by reducing ROS. PON2 can inhibit LDL oxidation and enhance the antioxidant properties and cholesterol efflux capacity of HDL, even though it is not found on lipoproteins [16,110,112,128]. Approximately 1600 molecules of cholesterol ester and 170 molecules of triglyceride reside in the central core of LDL particles [129]. Half of the fatty acids inside LDL are polyunsaturated fatty acids (PUFAs) that are mostly composed of linoleic acid but also include arachidonic acid and docosahexaenoic acid. All of these PUFAs are usually protected against free radical attack and oxidation by alpha-tocopherol and other antioxidants [130]. Whenever there is an imbalance in the levels of antioxidants and the amount of PUFAs, LDL is oxidized. LDL can be oxidized by metal ions, lipoxygenases, myeloperoxidase, and reactive nitrogen species, mainly under the aorta intima; this process is mediated by the cells residing in the aorta wall [131]. Oxidized LDL (OxLDL) plays a pivotal role in triggering proinflammatory events that initiate and exacerbate atherogenesis [132,133]. Cells overexpressing PON2 are less prone to oxidize LDL showing significantly less intracellular oxidative stress when exposed to either H_2_O_2_ or oxidized phospholipids [15], suggesting that PON2 plays a protective role in atherosclerosis. Indeed, when PON2-knockout and apoE null mice were challenged with a high-fat diet, those mice developed significantly larger atherosclerotic lesions than their wild-type counterparts. The serum levels of VLDL and LDL cholesterol were significantly lower in PON2-deficient mice compared with wild-type mice. Enhanced inflammatory signaling by LDL, an attenuated antiatherogenic capacity of HDL, and a heightened state of oxidative stress, along with an exacerbated inflammatory response in PON2-deficient macrophages, were also detected in the PON2-deficient mice [16,97,112]. Conversely, adenoviral overexpression of PON2 in apoE null mice significantly enhances the efflux potential and antioxidant capacity of serum and increases the anti-inflammatory properties of HDL, thus protecting mice against atherogenesis in vivo [111]. Further investigation showed that the antiatherogenic effects of PON2 are partly contributed by its protection against oxidative stress in mitochondria [2]. PON2 can prevent mitochondrial superoxide formation and apoptosis of cells, which is independent of its lactonase activity [1]. These studies are sufficient to show that PON2 strongly inhibits the development of atherosclerosis.

### 6.2. PON2 and Cancer

Growth and metastasis of a tumor depend on several factors but at least a relationship between inflammation, oxidative stress, and cancer has been demonstrated. Cancer cells display accelerated metabolism that can result from different mechanisms such as the increased activity of specific metabolic pathways, alterations of mitochondria and peroxisome functions, increased cellular receptor signaling, enhanced functioning of inflammatory cytokines, and activation of oncogenes [134,135,136,137]. During the initiation stage, ROS may contribute to oxidative modifications and impairment of biomolecules including DNA, polyunsaturated fatty acids of lipid membranes, and proteins. Oxidative alterations of proteins may result in the loss of enzyme activity and may render proteins more susceptible to proteolytic degradation [135,137,138]. ROS can also contribute to abnormal gene expression, impaired intercellular communications, and modifications of signaling pathways. In particular, ROSs activate stress-responsive survival pathways and can sustain cellular proliferation and differentiation [135,139,140]. Pathways that are activated by ROSs involve several enzymes such as p38 mitogen-activated protein kinase (p38 MAPK), protein kinase C (PKC), extracellular signal-regulated kinase (ERK)1/2, Jun N-terminal kinase (JNK), and phosphoinositide 3- kinase/serine-threonine kinase (PI3K/Akt) [139,140]. ROS are also involved in the regulation of transcription factors such as activator protein 1 (AP-1), the nuclear factor erythroid 2-related factor 2 (Nrf2), hypoxia-inducible transcription factor 1a (HIF-1a), p53, and nuclear factor κB (NF-κB) [140]. The antioxidant and antiapoptotic activities of PON2 suggested a role favoring cancer cell survival and chemotherapeutic resistance [4,141]. In fact, despite the established and prevailing role of paraoxonases in cardiovascular diseases and relevant parameters, more recent studies revealed an emerging association of PONs with cancer [142]. Microarray studies led to observe overexpression of PON2 in some solid tumors, like hepatocellular carcinoma, prostate carcinoma [143,144], and several others such as skin neoplasm [145], gastric cancer [146], and breast cancer [147]. Additionally, in various leukemia gene expression profiling studies, upregulation of PON2 could be demonstrated; an example is pediatric acute lymphoblastic leukemia (ALL) [148]. Importantly, a subsequent study identified PON2 as a member of a very small group of upregulated genes that characterized pediatric ALL patients with very poor outcome prognosis [149]. In another form of leukemia, chronic myeloid leukemia (CML), PON2 was also identified in an outcome-specific gene expression signature of primary imatinib-resistant patients [150]. Moreover, a marked overexpression of PON2 was observed in lymphocytes infected with T-cell leukemia virus [151]. In addition to the listed microarray data, Witty et al. (2011) recently showed that PON2 protein level is increased in some tumors. PON2 overexpression was detected in the pancreas, liver, kidney, and lung tumors and an over 10-fold upregulation of PON2 in thymus tumors and non-Hodgkin’s lymphomas [4]. PON2 is 2–4-fold overexpressed in the tumors from urinary bladder, liver, kidney, lymphoid tissues, and endometrium/uterus in comparison to normal tissue [4]. Despite some other tissues, where no increase in the expression level was observed, human tumors of the thyroid gland, testis, prostate, and pancreas showed a slight upregulation of PON2. Other studies about the relation of PON2 and cancer demonstrated that PON2 contributes to the progression and metastasizing of pancreatic cancer by stimulating glucose uptake [56], accelerates the proliferation of and resistance to oxidative stress in bladder cancer [152], protects glioblastoma cells against apoptosis [153], and reduces the sensitivity of oral cancer cells to radiation therapy [60]. An interesting phenomenon is that both PON2 and PON3 are upregulated in the early stages and some subtypes of cancer, whereas they are downregulated in the late stages. This could indicate that, especially in the early stages of tumor formation, the antioxidative and antiapoptotic function of PON2 and PON3 are beneficial as it helps to generate the platform for malignant transformation. This observation could represent a potential approach of innovative therapies trying to normalize the otherwise overexpressed PONs. In support to this hypothesis, the same study [4] revealed that knockdown of endogenous PON2 caused spontaneous apoptosis of several human cancer cell lines—an intriguing but somewhat unexpected finding given the viability of PON2-deficient mice (the residual PON2 expression in these mice [112] may be comparable to efficient cell culture RNAi experiments). An exciting question is how tumors achieve an increase in PON2 and/or PON3 expression. One simple explanation could be that, in some tissues, for example, papillary renal cell kidney carcinoma or prostate adenocarcinoma, chromosome 7, which contains the PON cluster, is amplified [144]. Another reason might be that the regulation depends on several signaling pathways, which are linked to reactive oxygen species and cancer, for example, PPAR-γ, AP-1, β-catenin/Wnt, NF-κB, HIF-1α, PI3K, and Nrf2 [138]. In accordance, earlier studies showed that PON2 expression is enhanced by oxidative stress [64], PI3K/PDGFR, PPARγ, and NADPH oxidase activation and by AP-1 activation [67,154]. Another point of interest is why some tumors upregulate PON2 or PON3. It is known that one hallmark of cancer is resistance to cell death [155] and paraoxonases 2 and 3 provide a protection against mitochondrial cell death signaling [4,156]. Their overexpression lowered susceptibility to different chemotherapeutics (e.g., imatinib, doxorubicin, and staurosporine) in cell culture models via diminishing proapoptotic mitochondrial O_2_-formation. Oxidative stress and chronic inflammation are closely linked to cell death and cancer [138], therefore it appears conceivable that tumors take advantage of the antioxidative function of PON2/PON3 to escape cell death. Other mechanisms potentially involved in the antiapoptotic role of PON2 in tumor cells include UPR, a signaling pathway triggered during cell stress. In fact OSCC cells overexpressing PON2 were protected against UPR-mediated cell death [157]. A lower activity of JNK and reduced expression of the proapoptotic factor CHOP related to overexpression of PON2 has been demonstrated. These changes culminate in a decreased activation of caspase [4]. In a 2018 study for the first time, it was analyzed the expression level and mutations of PON2 gene in 31 types of malignancies and investigated the association between the expression level of PON2 and patient survival. The results confirmed that the highest level of PON2 expression is observed in solid tumors, in particular in brain tumor and liver cancer. Amplification of the PON2 gene and correlation of its expression with unfavorable prognosis of survival were also typical of these tumors. Moreover, PON2 located in the nuclear envelope and endoplasmic reticulum could protect cancer cells against unfavorable environmental conditions and against chemotherapy. Patients with glioblastoma, low grade glioma, liver hepatocellular carcinoma, and acute myeloid leukemia, exhibiting higher expression of PON2, had poor survival when compared with patients with lower PON2 expression [6]. There is only one study in which it is demonstrated that PON2 acts as a tumor suppressor. In the early stage of OC by reducing IGF-1 production and signaling, PON2 activation might be a fruitful strategy to inhibit early-stage ovarian tumor. ID8hPON2 cells overexpressing human PON2 developed reduced tumors. Based on these data PON2 overexpression regulates the antitumorigenic pathways. I). PON2 overexpression reduces mitochondrial superoxide levels, which regulate c-Jun activation. Reduced c-JUN binding to the IGF-1 promoter leads to decreased expression of IGF-1 protein. II). PON2 expression enhances electron transport chain activity leading to decreased cholesterol levels resulting in impaired caveolin-1 and IGF-1R interaction. I and II result in decreased cell proliferation and reduced tumor growth [158].

### 6.3. PON2 and Insulin Sensitivity

Atherosclerosis and insulin resistance are multifactorial diseases, which commonly associate with dyslipidemia, oxidative stress, obesity, hypertension, and chronic inflammation. The liver is not only the primary site of lipid metabolism, but is also the primary site for glucose uptake, production, and storage. Systemic and local oxidative and inflammatory stimuli greatly influenced its role in glucose metabolism [159,160], which in turn influences whole-body insulin responsiveness [161]. Given the elevated oxidative stress levels and the abnormal lipid metabolism, previously reported in PON2-deficient mice [2,112], Bourquard et al. hypothesized atherosclerosis as accompanied by impaired hepatic insulin signaling and showed PON2 deficiency as associated with inhibitory insulin-mediated phosphorylation of hepatic insulin receptor substrate-1 (IRS-1) [107]. Factors secreted from activated macrophage cultures derived from PON2-deficient mice are sufficient to modulate insulin signaling in cultured hepatocytes like that observed in vivo. The modulation of hepatic insulin sensitivity by PON2 seems mediated by a shift in the balance of NO and ONOO− (peroxynitrite) formation. These studies show that PON2 plays a pivotal role in insulin sensitivity by its ability to modulate reactive species most likely as a result of PON2’s association with the mitochondrial function [141].

From the previous data, it is clear that PON2 modulates the execution of the cell death program directly at the mitochondria, thereby allowing modification of several apoptotic pathways converging at these organelles. Given the importance of ER stress, JNK signaling, and CHOP expression for b-cell homeostasis [162], PON2 may impact on diabetes.

### 6.4. PON2 and Neurodegeneration

PON2 is unique among PONs to be expressed in the brain tissue. Due to its cellular localization and antioxidant and anti-inflammatory actions may represent a relevant enzyme involved in neuroprotection. PON2 levels are highest in dopaminergic regions (i.e., striatum), where oxidative stress is higher due to dopamine metabolism. Levels are higher in astrocytes than in neurons and are even higher in the brain and peripheral tissues of female mice than male mice. The regional distribution and gender differences of PON2 were confirmed by measurements of its lactonase activity (measured by dihydrocoumarin (DHC) hydrolysis) and of PON2 mRNA levels [163]. Its differential expression in males and females may explain gender differences in the incidence of various diseases, including neurodevelopmental, neurological, and neurodegenerative diseases. Lack of PON2 (as in PON2−/− mice) or lower levels of PON2 (as in male mice compared to females) increases susceptibility to oxidative stress-induced toxicity. Estradiol increases PON2 expression in vitro and in vivo and provides neuroprotection against oxidative stress. Such neuroprotection is not present in CNS cells from PON2−/− mice [100]. PON2 mRNA has been observed in mouse and human brains [8,10,96,164] and PON2 protein has been detected in mouse [97,111] and monkey brains [102]. In a series of recent studies, the expression of PON2 has been characterized in the mouse brain [163,165]. In the brain, and to a lesser extent in kidney and testis, but not in all tissues, the PON2 antibody recognized two bands, the lower at Mw 43 kDa, which corresponds to the reported Mw of PON2, and an upper band at Mw 55 kDa. This last band had been found at times by some investigators [3,4,79,97,166], but not by others [14,98,99,154], and has been suggested to be a PON2 alloform, following the two mRNA splicing variants [3,10,79]. In our recent study in HeLa cells [23], we identified five different bands. By immunoprecipitation and MS analysis, we found that the 40 kDa band is the truncated version of PON2 lacking the peptide ranging from residues 123 to 134 (isoform 2), whereas the 43 kDa band contains both the canonical full-length protein (isoform1) and a modified version (differences in the first 16 amino acids) called Primo Parmo (P.P.) isoform first described by Primo Parmo et al. [8,23]. PON2 is known to have four putative N-linked glycosylation sites at asparagine residues [19] but experiments of site-directed mutagenesis and deglycosylation indicated that both putative isoforms are glycosylated and only on N254 and N323. Nevertheless, neither band was detected in the brain from PON2-deficient mice [163]. Several studies reported the association of PON2 [37,167,168,169] and of codon 311 (S > C) PON2 polymorphism [34,35,46] with Alzheimer’s disease (AD). PON2 neuroprotection against oxidative stress was also suggested [102]. Polymorphisms in PON1 have also been associated with susceptibility to Parkinson’s disease [170,171]. The Parkinson’s disease (PD) is a progressive neurodegenerative disorder characterized by the selective loss of the pigmented dopaminergic neurons of the substantia nigra pars compacta (SNpc) [172], and the reduction in the striatal dopamine level. The majority of PD cases do not follow a genetic inheritance pattern [173]. However, rare familial forms of this disease with their causative genes have been defined [174,175,176,177]. DJ-1 was identified as one of these PD-related genes, and it has been most associated with the management of ROS, even if it is not completely clear how DJ-1 may regulate ROS [178,179]. In a proteomic interaction screen for DJ-1 interacting partners, PON2 was reported as a novel interacting candidate for DJ-1 [179]. It has been shown that DJ-1 interacts with PON2 in neurons and cell lines. This interaction appears to modulate PON2 activity as DJ-1 KO cells have less basal PON2 activity and do not respond to oxidative stress as DJ-1 WT cells do. This effect can be reversed by the expression of DJ-1. Besides, the expression of PON2 in DJ-1 KO neurons is more protective against the Parkinson’s model of neuronal death than the expression of DJ-1 in PON2 deficient background. In summary, DJ-1 interacts with and promotes PON2 activity in the presence of oxidative stress and this mechanism is one central mechanism by which DJ-1 promotes cell survival [180].

In the context of PD, neuronal death is determined by many factors that include ER and oxidative stress. Tissue samples from PD patients show increased P-PERK, P-eIF2α, and ATF4 in the SNpc [181]. Another study showed that the DA neurotoxin mimicking PD, 1-methyl-4-phenylpyridinium ion (MPP+) and the neurotoxin 6-hydroxydopamine (6-OHDA) widely used to induce models of PD upregulate the expression of UPR proteins such as BIP, CHOP, ATF4, P-PERK, and P-eIF2α [182]. DJ-1 null neurons are hypersensitive to ROS yet show remarkable resistance to ER stress. The potential role of ER stress in PD is growing. Hyperactivation of UPR is observed in PD patients and animal models. However, the contribution of ER stress pathways to neurodegeneration is complex, with the involvement of both prosurvival and prodeath pathways. For example, a dual role of ATF4 has been shown during ER stress. ATF4 translation is upregulated through increased P-eIF2α, in response to the accumulation of misfolded proteins in the ER. This event prevents mitochondrial damage by promoting the transcription of Parkin. However, induction of ATF4 can also lead to cell death through CHOP induction and caspase-3 activation. DJ-1 regulates the level of ATF4 in response to ER stress. This evidence is summarized as follows: (1) DJ-1 loss reduces basal and ER stress-induced ATF4 induction. (2) Expression of WT or mutant DJ-1 leads to increased ATF4 levels. (3) ATF4 regulation is not due to DJ-1 transcriptional activity. Instead, DJ-1 directly binds ATF4 transcripts increasing its stability. Surprisingly, DJ-1 loss is associated with neuronal survival under acute ER stress. This observation runs counter to most reports of DJ-1 acting as a prosurvival factor [181]. In a recent study [183] it was also demonstrated that the β-estradiol-3-benzoate (EB) has a protective effect on the neurodegenerative experimental model of Parkinson’s disease. The protective effect is through the induction of the expression of paraoxonase-2 (PON2) in the striatum. PON2 in fact has antioxidant and anti-inflammatory activity, with a beneficial effect in the MPP+ model in rats decreasing the lipid peroxidation and the oxidative stress [183]. It is interesting to note that in the last decade several papers associated PON2 and its polymorphisms also with amyotrophic lateral sclerosis (ALS) [184,185]. ALS is a multifactorial disease characterized by cerebral cell dysfunction and mitochondrial alteration. It is associated with the progressive increase in neuroinflammation, generalized oxidative stress, and metabolic alterations [186,187]. Saeed et al. [188] identified high linkage disequilibrium in PON2 and PON3 genes. The C allele of the C311S PON2 and R allele of the Q192R PON1 polymorphism were instead associated with sporadic ALS. The presence of the R-C haplotype has been linked to the development of ALS [189,190]. A SNP haplotype was found in the C-terminal portion of PON2 that included the alteration of amino acids PON2 C311S in the French and Canadian population, and in the other combined populations. The casuistic stratification showed that this alteration was a relevant risk factor for the development of ALS, regardless of the patient’s nationality [191]. In addition, seven mutations found in the PON gene in patients with familial and sporadic ALS were observed [192]. However, in an Italian population, the SNPs L55M, Q192R in PON1, and C311S in PON2, both genotype and haplotype, were not associated with ALS [193]. Furthermore, in a study with nine polymorphisms present in the PON gene cluster, including rs7493 (G > C) and rs11981433 (T > C, G) in the PON2 gene in the Chinese population, no association was found between these SNPs and sporadic ALS [194]. In addition, the expression of messenger RNA of the PON2 gene was decreased in the spinal cord and trunk tissue of patients with ALS and PON1 was undetectable [195].

## 7. Conclusions and Perspectives

The emerging picture of the last few years is that PON2 is placed in different districts and exert different functions: (1) on the plasma membrane PON2 represents the first line of defense against infections by hydrolyzing 3OC12HSL [25]; (2) it has antiatherogenic effects exerted by removing peroxidized lipids from the membrane [21]; and (3) connection of PON2 with cancer for its antiapoptotic roles are emerging [141]. In the public databases are reported three isoforms of PON2 protein derived from splice variants of mRNA [3,10,79] and recently it was reported the first evidence of the isoform 1 P.P. and isoform 2 as expressed proteins [23]. PON2 is rapidly inactivated by ubiquitination induced after incubation of cell extracts with 3OC12HSL [22] and it is ubiquitinated and ADP-rybosilated under physiological conditions (without 3OC12HSL) in intact cells [23]. PON2 PTMs cluster nearby the two polymorphic sites 148 and 311 that have been previously implicated in diabetes and cardiac failures [36,51,53]. It has been demonstrated, in vitro, that these SNPs are indeed involved in the PON2 activity and that PTMs nearby could be involved in modulating PON2 activity [23]. Contrasting evidence about the effect on activity of S311C mutation were reported [1,19] and the data of Stoltz et al. [19] that reported lower activity of the mutant (Cys/Cys alleles) expressed in CHO cells and decreased lactonase activity in primary airway epithelial cells were confirmed by Carusone et al., 2020 [23]. Since the 311 site is nearby position 313 that was found ubiquitinated [69], it is tempting to speculate that both these PTMs affect PON2 activity. The interesting data about the effect of the SNPs on PON2 catalytic activity and the identification of PTMs near these sites, require the knowledge of PON2 structure to link the ubiquitination, the consequent modulation of catalytic activity and the SNPs role in a single picture. An mRNA operon including at least PON2, BIRC3, WTAP, and WDR36 was hypothesized and confirmed [23]. The operon could work through the control mediated by WTAP of the alternative splicing of its own and controlled genes. BIRC3 inhibits the formation of the PON2 Isoform 2 that previous studies confirmed to encode a disordered structure, in agreement with the fact that it is 95% inactive in vitro (unpublished data). The proposed model (Figure 6) for PON2 post-transcriptional regulation is just the starting point of a picture in which different genes take part by protein–protein interactions and more intricate signaling pathways.

Below are reported compounds and molecules able to modulate PON2 expression and/or function. Arachidonic acid [196], unesterified cholesterol, pomegranate juice [154], the licorice phytoestrogen glabidrin [196], and the hypocholesterolemic drug atorvastatin [197] upregulate PON2 expression in various cell types. In mouse fibroblasts, dexamethasone increases PON2 mRNA levels [198]. Quercetin was reported to increase PON2 mRNA and protein in macrophages in vitro [199]. The latter was confirmed in mouse astrocytes [165]. Extracts of *Yerba mate* (*Ilex paraguariensis*) have been reported to increase PON2 mRNA and lactonase activity in macrophages in vitro and after in vivo administration to healthy women [200]. Early Glycated end products such as glycated albumin (GA) and advanced glycated end products such as Nε-(carboxymethyl) lysine (CML) downregulate PON2 expression and activity in human umbilical vein endothelial cells (HUVECs) [201].

Recent papers suggest the involvement of PON2 on more diseases than previously anticipated, such as cataract [40], COVID-19 [125] ischemic stroke [202], and noise-induced hearing loss [203,204]. In Table 1 we collected the most representative works for each related disease and we discriminated between PON2 wildtype and PON2 polymorphisms association. From the table is clear that cancer studies are the most recent and that cancer is never associated with polymorphisms. This can be explained by the fact that PON2 is involved in cancer for its antiapoptotic and antioxidant roles while its polymorphisms affect mainly the lactonase activity.

According to these effects further studies will allow one to identify the missing pieces and the full knowledge of PON2 functions and mechanisms in human cells that in turn will provide an innovative therapeutic target based on the increase or decrease of PON2 expression in relation to the associated diseases.

## Figures and Tables

**Table 1:** PON2 and related human diseases.

	Disease	References	
**Heart Diseases**			
**PON2**	Atherosclerosis	Devarajan, A., et al., 2011	[2]
		Ng, C.J., et al., 2001	[14]
		Ng, C.J., et al., 2005	[15]
		Ng, C.J., et al., 2006a	[97]
		Ng, C.J., et al., 2006b	[112]
	Acute myocardial ischemia	Marchegiani, et al., 2009	[42]
**S311CSNP**		Sulaiman, D., et al., 2019	[103]
	Coronary Artery Disease (CAD)	Li, W., et al., 2018	[5]
**PON2 SNPs**		Wang, X., et al., 2003 Martinelli, N., et al., 2004	[31] [32]
		Sanghera, D.K., et al., 1998	[33]
		Shin, B.S., et al., 2008	[38]
		Chen, Q., et al., 2003	[39]
		Chen, M., et al., 2016	[41]
		Leus, F.R., et al., 2001	[45]
	Large vessel disease	Slowik, A., et al., 2007	[43]
**PON2SNP**	Covid-19 (blood coagulation)	Liu, D., et al., 2020	[125]
**Metabolic Diseases**			
**PON2**	Insulin sensitivity	Qujeq, D., et al., 2018	[50]
		Bourquard, N., et al., 2011	[107]
**PON2** **PON2SNPs**	Diabetes complications	Pinizzotto, M., et al., 2001	[49]
		Mackness, B., et al., 2005	[51]
**Cancer**			
**PON2**	Different types of malignancies	Witte, I., et al., 2011	[4]
		Shakhparonov, M.I., et al., 2018	[6]
		Witte, I., et al., 2012	[141]
		Bacchetti, T., et al., 2019	[142]
	Hepatocellular carcinoma	Yao, L., et al., 2002	[143]
	Prostate carcinoma	Ribarska, T., et al., 2010	[144]
	Skin neoplasms	Bacchetti, T., et al., 2020	[145]
	Gastric cancer	Wang, X., et al., 2019	[146]
	Breast cancer	Wang, R., et al., 2018	[147]
	Pediatric acute lymphoblastic leukemia (ALL)	Ross, M.E., et al., 2003 Kang, H., et al., 2010	[148] [149]
	Chronic myeloid leukemia	Frank, O., et al., 2006	[150]
	Human T-cell leukemia virus 1	Pise-Masison, C.A., et al., 2002	[151]
	Pancreatic cancer	Nagarajan, A., et al., 2017	[56]
	Bladder cancer	Bacchetti, T., et al., 2017	[152]
	Glioblastoma	Tseng, J.H., et al., 2017	[153]
	Oral squamous cell carcinoma (OSCC)	Krüger, M., et al., 2016 Krüger, M., et al., 2015	[60] [157]
	Ovarian tumor	Devarajan, A., et al., 2018	[158]
**Neurodegenerative diseases**			
**PON2**	Neuroprotection	Costa, L.G., et al., 2014	[102]
	Alzheimer	Shi, J.; et al., 2004	[35]
		Erlich, P.M., et al., 2006	[46]
		Nie, Y., et al., 2017	[167]
		Leduc, V., et al., 2011	[168]
		Mu, N., et al., 2013	[169]
		Janka, Z., et al., 2002	[34]
**PON2 SNPs**	Parkinson	Parsanejad, M., et al., 2014	[180]
		Aguirre-Vidal, Y., et al., 2020	[183]
	ALS	Narain, P., et al., 2019	[185]
		Saeed, M., et al., 2006	[188]
		Landers, J.E., et al., 2008	[190]
		Valdmanis, P.N., et al., 2008	[191]
		Ticozzi, N., et al., 2010	[192]
		Ricci, C., et al., 2011	[193]
		Chen, Y.P., et al., 2012	[194]
**Other diseases**			
**PON2 SNPs**	Cataract	Baig, A., et al., 2019	[40]
**PON2**	Ischemic stroke	Rodríguez-E, F., et al., 2017	[202]
**PON2 SNPs**	Noise-induced hearing loss	Wu, S.S., et al., 2020	[203]
		Li, X., et al., 2016	[204]
